# Animal Models in Regulatory Breakpoint Determination: Review of New Drug Applications of Approved Antibiotics from 2014–2022

**DOI:** 10.3390/jpm14010111

**Published:** 2024-01-19

**Authors:** Daniel Selig, Diana Caridha, Martin Evans, Adrian Kress, Charlotte Lanteri, Roseanne Ressner, Jesse DeLuca

**Affiliations:** Walter Reed Army Institute of Research, Experimental Therapeutics, Silver Spring, MD 20910, USA; diana.caridha2.ctr@health.mil (D.C.); martin.o.evans.mil@health.mil (M.E.); adrian.t.kress.mil@health.mil (A.K.); charlotte.a.lanteri.mil@health.mil (C.L.); roseanne.a.ressner.mil@health.mil (R.R.);

**Keywords:** animal models, antibiotics, breakpoint determination, clinical pharmacology, Food and Drug Administration

## Abstract

We sought to better understand the utility and role of animal models of infection for Food and Drug Administration (FDA)-approved antibiotics for the indications of community-, hospital-acquired-, and ventilator-associated bacterial pneumonia (CABP, HABP, VABP), complicated urinary tract infection (cUTI), complicated intra-abdominal infection (cIAI), and acute bacterial skin and structural infections (ABSSSIs). We reviewed relevant documents from new drug applications (NDA) of FDA-approved antibiotics from 2014–2019 for the above indications. Murine neutropenic thigh infection models supported the choice of a pharmacokinetic-pharmacodynamic (PKPD) target in 11/12 NDAs reviewed. PKPD targets associated with at least a 1-log bacterial decrease were commonly considered ideal (10/12 NDAs) to support breakpoints. Plasma PK, as opposed to organ specific PK, was generally considered most reliable for PKPD correlation. Breakpoint determination was multi-disciplinary, accounting at minimum for epidemiologic cutoffs, non-clinical PKPD, clinical exposure-response and clinical efficacy. Non-clinical PKPD targets in combination with probability of target attainment (PTA) analyses generated breakpoints that were consistent with epidemiologic cutoffs and clinically derived breakpoints. In 6/12 NDAs, there was limited data to support clinically derived breakpoints, and hence the non-clinical PKPD targets in combination with PTA analyses played a heightened role in the final breakpoint determination. Sponsor and FDA breakpoint decisions were in general agreement. Disagreement may have arisen from differences in the definition of the optimal PKPD index or the ability to extrapolate protein binding from animals to humans. Overall, murine neutropenic thigh infection models supported the reviewed NDAs by providing evidence of pre-clinical efficacy and PKPD target determination, and played, in combination with PTA analysis, a significant role in breakpoint determination for labeling purposes.

## 1. Introduction

Animal models of disease are an essential tool to screen for the safety and efficacy of compounds, while allowing informed projections for human use [1]. Selection of appropriate animal models and endpoints may be critical to the success of a translational drug development program [2]. For anti-bacterial agents, the importance of selecting appropriate animal models and endpoints is underscored by a recent FDA workshop and multiple FDA guidance documents [3,4,5]. In addition to animal survival data, the FDA suggests exploring PKPD relationships and organ-specific PK, while considering protein binding and translatability to humans [3,4,5].

Two of the most cited animal models utilized for the investigation of antibiotics are murine neutropenic thigh infection and murine neutropenic lung infection models [6,7]. These models allow for the characterization of in vivo survival and the optimal PKPD index, which may be used to provide reliable projections for human efficacy [7,8]. Once the optimal PKPD index is established, population PK (popPK) modeling in combination with allometric scaling and probability of target attainment analysis (PTA) could be used to define an ideal range of doses to test in first-in-human (FIH) studies [9]. This becomes an iterative process where PK and safety data from the FIH studies can further refine the popPK modelling process and inform dose selection for more advanced phases of drug development [10]. Sparse PK sampling embedded in larger trials allows for the continuation of this iterative model-informed drug development (MIDD) process, further improving the popPK model, allowing for exposure-analysis responses and potentially providing support for dosing in special patient populations such as those with impaired kidney function [11].

However, despite robust pre-clinical and clinical data in combination with MIDD, an optimal breakpoint determination for novel antibiotics remains challenging. Breakpoint determination is a multi-disciplinary process that incorporates cutoffs derived from pre-clinical PKPD data, clinical efficacy and exposure-response data, and epidemiologic wild-type cutoffs [12]. Multiple stakeholders collaboratively determine the final breakpoint given the available cutoff data. Importantly, different scenarios and stakeholder biases may lead to the heightened or lowered importance of specific cutoff data [12,13,14]. For example, gathering sufficient clinical data to precisely estimate a clinical cutoff may not be feasible. This may be in part due to tendencies for registration trials not to include patients most likely to be infected with the most resistant pathogens and generally low sample sizes of patients with infections caused by pathogens at higher MICs [12,15]. In some instances, as commented by FDA clinical pharmacology review teams in ceftazidime-avibactam and meropenem-vaborbactam reviews, registration trials may demonstrate such high rates of antibiotic efficacy and/or achieving pre-defined PKPD targets that exposure-response analyses are not feasible [16,17]. In such cases, clinical cutoffs may be significantly less useful in determining the overall breakpoint.

Given these complexities of breakpoint determination, we sought to better understand the role of cutoffs derived from pre-clinical PKPD animal studies in the final breakpoint determination for newly approved FDA antibiotics. In addition, we sought to gain insights on the general utility of animal models for the PKPD index determination and how this may be used for off-label dosing in situations where clinical data may be lacking.

## 2. Materials and Methods

We reviewed the FDA clinical pharmacology or multi-disciplinary reviews for antibiotics approved from 2014–2022 for the indications of CABP, HABP, VABP, cUTI, cIAI and ABSSSI. Such reviews are available to the public and may be generally found at the Drugs@FDA website: https://www.accessdata.fda.gov/scripts/cder/daf/index.cfm (accessed on 14 November 2023). Specific citations to the documents and access dates may be found in the references section. The selection of an optimal PKPD index may play a significant role in PTA success and hence breakpoint determination [18]. Therefore, to better understand the decision-making process of optimal PKPD index determination, we extracted PKPD indices against the most clinically relevant pathogens for the animal models cited in the review documents. In addition, we extracted data on protein binding, epidemiologic cutoffs, PKPD-based cutoffs, clinical cutoffs and whether the sponsor and FDA agreed on the final breakpoint determination. When an antibiotic was approved for more than one indication, we presented data from the indication with the most conservative measures of PKPD index and breakpoint. We generally limited the data extraction to the most virulent pathogens. Such pathogens are commonly targeted in clinical practice, and generally, if breakpoints for virulent pathogens are achievable, it follows that breakpoints for less virulent pathogens should also be achievable. In addition, the breakpoints listed only represent the initial breakpoint determined by the FDA at the time of approval. The revision of breakpoints is a separate topic and has its own considerations [19]. A comprehensive list of FDA-recognized breakpoints by indication and pathogen may be found at https://www.fda.gov/drugs/development-resources/antibacterial-susceptibility-test-interpretive-criteria (accessed on 14 November 2023).

## 3. Results

From 2014–2022, there were 13 antibiotic reviews available from Drugs@FDA with clinical pharmacology or multi-disciplinary reviews. Relevant data were extracted from 12 of 13 reviews, as the eravacycline NDA filing did not include sufficient PKPD data to make strong conclusions about breakpoints in relation to the use of animal models [20]. Eight of the remaining twelve antibiotics were novel compounds [21,22,23,24,25,26,27,28], and four of the remaining twelve were previously approved antibiotics in combination with a beta-lactamase inhibitor [16,17,29,30]. Antibiotics were approved for a broad range of indications, including four approvals for ABSSSI single indication [21,22,23,24], one approval for cUTI single indication [17], one for CABP single indication [28], with the remaining six being approved for multiple indications, including combinations of cUTI, cIAI, ABSSSI and HABP/VABP. 

Table 1 summarizes the animal models, PKPD indices, and clinical PKPD correlations extracted from the regulatory documents that were used to support breakpoint decision-making. Regardless of the indication, all regulatory filings except for Lefamulin (11/12) cited dose fractionation studies with a neutropenic thigh model. The Lefamulin (approved solely for the indication of CABP) review cited only a murine pneumonia model of infection. Omadacycline, cefiderocol, ceftazidime-avibactam and plazomicin regulatory filings cited both murine thigh and murine pneumonia models of infection. Of note, ceftazidime-avibactam and plazomicin did not receive approval for pneumonia indications, whereas omadacycline and cefiderocol did receive approval to treat CABP and HABP, respectively.

Data generated from neutropenic thigh infection models were used to inform the selection of optimal PKPD indices in 10/12 regulatory filings [16,17,21,22,24,25,26,27,29,30]. Tedizolid and lefamulin were the only exceptions, where the optimal PKPD index for tedizolid was the bacteriostatic target derived from a non-neutropenic thigh infection model. The optimal index for lefamulin was the 1-log kill target determined from a neutropenic lung infection model. The final decision on the optimal PKPD index was arbitrary. For example, 9/10 antibiotics deriving PKPD indices from a neutropenic thigh infection incorporated a 1-log kill target into the decision-making process [16,17,22,24,25,26,27,29,30]. Of these antibiotics, 5/9 regulatory filings also considered the static target as possibly optimal [21,22,24,26,30]. The filing for dalbavancin cited static and 2-log kill, but not 1-log kill, targets [21], whereas the filing for meropenem-vaborbactam cited 1-log and 2-log kill targets, but not stasis [17]. 

When adequate PKPD data were available from the large registration trials, clinically derived PKPD targets were also considered in breakpoint determination. For five antibiotics, stratified outcomes by MIC [28,29] or exposure-response analysis [21,22,27] supported breakpoint determination. Clinical trials from the remaining six antibiotics did not have adequate data for formal exposure-response or outcome stratification by MIC [16,17,23,24,26,30]. Therefore, at the time of the regulatory filing, a clinical breakpoint determination was not feasible for these antibiotics. Common reasons cited in the regulatory filings were the lack of infections caused by bacteria with higher MICs and very high cure rates (>80–90%) against bacteria at all MICs observed in the trials. Data from omadacycline trials were insufficient for clinical breakpoint determination; however, the cure rates observed in patients with infections with high MICs supported the overall breakpoint, in addition to data from pre-clinical infection models [25].

Table 2 summarizes the epidemiologic cutoffs, PKPD cutoffs determined from pre-clinical and clinical data, the overall proposed breakpoints and the agreement of applicant and FDA proposed breakpoints. Generally, the epidemiologic cutoffs were consistent with cutoffs determined from clinical and pre-clinical data. The overall proposed breakpoint was always a multi-disciplinary decision considering all available cutoffs and clinical data. Where clinical cutoffs were not possible to obtain, the overall proposed breakpoint was largely influenced by the nonclinical PKPD cutoff. In most cases, the proposed breakpoint was equivalent to the nonclinical PKPD cutoff. For cefiderocol, however, the proposed breakpoints were half of the nonclinical PKPD cutoff, which was driven by a lack of observed clinical cure at higher MICs. Of the pathogens reviewed, FDA and applicant proposed breakpoints were in agreement for five antibiotics [17,22,23,24,30], disagreement for two antibiotics [21,28], and for five antibiotics this information was not available [16,25,26,27,29]. When FDA and applicant proposed breakpoints did not agree, FDA recommended lower breakpoints for *S. aureus* than the dalbavancin applicant [21]. For lefamulin, although the review states that the FDA did not agree with the applicant’s proposed breakpoints, the applicant’s breakpoints were not disclosed [28].

## 4. Discussion

We have reviewed 12 FDA clinical pharmacology reviews or multi-disciplinary reviews for antibiotics approved from 2014–2022. We found that the neutropenic thigh infection model supported most antibiotic NDAs for the purpose of breakpoint determination. In addition, our review highlights the high consistency of cutoffs derived from a combination of nonclinical PKPD targets in combination with PTA with epidemiologic cutoffs and clinical cutoffs. Overall, the neutropenic thigh infection model therefore has high utility as a tool to help inform clinical dosing decisions and support regulatory submissions.

A challenge consistently observed when reviewing the FDA documents was the lack of infections caused by pathogens with high MICs and still within the range of the estimated nonclinical cutoff. For example, PTA analysis for cefiderocol was adequate to support a breakpoint of 4 µg/mL against *P. aeruginosa*. However, there were limited clinical data for such isolates, in addition to lower overall success rates in patients with *P. aeruginosa* compared to other susceptible pathogens. Therefore, despite epidemiologic cutoffs and nonclinical cutoffs of 2 and 4 µg/mL for cefiderocol against *P. aeruginosa*, the final breakpoint was set at 1 µg/mL [27]. In other cases, such as for ceftazidime-avibactam where data was also lacking for isolates at higher MICs, the final proposed breakpoint against *P. aeruginosa* was 8 µg/mL. This was consistent with the epidemiologic cutoff of 4–8 µg/mL and was largely driven by the nonclinical cutoff of 8 µg/mL, in addition to adequate cure rates at the observed MICs in the trials [16].

An additional challenge in weighting the importance of nonclinical cutoffs in breakpoint determination is the applicability of the animal model to a human infection. Despite a large range of approved indications, nonclinical cutoffs from the neutropenic thigh infection model were generally consistent with epidemiologic cutoffs and clinical cutoffs when available. However, some antibiotics may not penetrate the lung as effectively as others [31] or may be inactivated in lung tissue, such as daptomycin [32]. Therefore solely relying on a neutropenic thigh infection model to determine breakpoints for non-ABSSI infections may lead to inadequate dosing decisions. 

Furthermore, the optimal PKPD index determination and relationship to breakpoints are not firmly established. There appears to be general agreement between nonclinical cutoffs and epidemiologic and clinical cutoffs when assuming 1-log kill targets from the neutropenic thigh models. However, some antibiotics achieved regulatory success when assuming static or 2-log kill targets [21,23]. Tedizolid, in particular, supported breakpoint determination using a non-neutropenic thigh model when assuming a bacteriostatic index. This bacteriostatic PKPD index, with an AUC24/MIC of 15, was 16.66 times lower than the static AUC24/MIC index from the neutropenic thigh model. Nevertheless, dosing based off the non-neutropenic thigh model in combination with PTA provided valuable metrics for dose prediction and breakpoint determination [23].

Other significant considerations from FDA reviewers were the translatability of protein binding between species and PKPD in relation to specific clinical outcomes. Regarding protein binding, lefamulin was found to have approximately 20–25% protein binding in a murine model. In comparison, protein binding in adult humans was 86–97% in vitro and 94.5–97.2% in vivo. The FDA discusses in this review that the FDA PTA and PKPD analyses assumed protein binding of 94–97%, whereas the applicant assumed protein binding of 73–88% for corresponding analyses [28].

Regarding PKPD in relation to clinical outcomes, dalbavancin has a long half-life of 1–2 weeks [21,33]. Early clinical response after 48–72 h is the FDA preferred endpoint for ABSSSI, whereas clinical response at end of treatment is considered secondary [21,34]. In determining clinical cutoffs, the dalbavancin applicant correlated a 5 day AUC average MIC ratio (AUCavg/MIC) to test of cure (14 days after therapy) [35]. The FDA performed multiple analyses but based the primary decision making on the PKPD analysis of AUCavg/MIC with the primary endpoint of early clinical response. As dalbavancin was dosed weekly and has a long half-life, drug exposure accumulates in the second week. Therefore, the correlation of AUCavg/MIC with the early clinical outcome represents both a stronger temporal relationship and likely a more accurate PKPD correlation as compared to a similar analysis for outcomes at test of cure. The FDA reviewer also noted that the predicted test-of-cure clinical success rate was significantly higher than predicted at the time of the early clinical outcome, and less than 1% of isolates of *S. aureus* in the studies had an MIC of 0.25 µg/mL. These points summarize the FDA reviewer’s rationale in selecting a dalbavancin breakpoint of 0.06–0.125 µg/mL as compared to the dalbavancin’s applicant proposed breakpoint of 0.25 µg/mL against *S. aureus*. 

Overall, the neutropenic thigh infection model serves as a strong pre-clinical model to help inform clinical efficacy, dose selection and breakpoint determination for a wide range of human infections. All data must be considered to determine optimal breakpoints. These data include, at minimum, epidemiologic cutoffs, nonclinical cutoffs, clinical cutoffs, the translatability of antibiotic properties such as protein binding from animals to humans and the general translatability of findings from the neutropenic thigh infection model to various human infections. 

## Figures and Tables

**Table 1:** PKPD indices to support breakpoint decision making.

Antibiotic	Year Approved	Labeled Indication(s)	Neutropenic Thigh	Pneumonia	Preclinical Target to Support Breakpoint	Clinical Target to Support Breakpoint
Ceftolozane-Tazobactam [29]	2014, 2019 additional approval for HABP/VABP	cIAI, cUTI, HABP/VABP	%T > MIC Ceftolozane *P. aeruginosa* Static: 24% 1-log kill: 31.5% 2-log kill: 52.2%		Neutropenic thigh 40%T > MIC	Clinical cure rates at various MICs.
Dalbavancin [21]	2014	ABSSSI	fAUC24/MIC: Static: 265 2-log kill: 332 Protein bound: 93%		Neutropenic thigh fAUC24/MIC static and 2-log kill	AUCavg/MIC of 13,396 where AUCavg is the mean AUC from day 1 and day 8 of treatment. Corresponds to 20% reduction in baseline area by day 4.
Oritavancin [22]	2014	ABSSSI	AUC72/MIC: Static: 3941 1-log kill: 4581		Neutropenic thigh AUC72/MIC stasis and 1-log kill	Comparison of AUC72/MIC for early clinical endpoint, 20% reduction in lesion by day 3 and at post-treatment evaluation.
Tedizolid [23]	2014	ABSSSI	Static AUC24/MIC Neutropenic: 250 Non-neutropenic: 15		Non-neutropenic thigh model bacteriostasis	Flat exposure-response relationship, where higher exposure was not associated with higher clinical response rates, limited the utility of clinical PKPD breakpoint determination.
Ceftazidime-Avibactam [16]	2015	cIAI, cUTI	Avibactam %fT > 1 mg/L Static: 40.2% 1-log kill: 50.3% Protein bound: 5.7–8.2% 50% fT > CAZ-AVI MIC	Avibactam %fT > 1 mg/L Static: 20.2% 1-log kill: 24%	Neutropenic thigh 1-log kill	Exposure-response analyses of individual exposures and microbiologic outcomes in Phase II cIAI and cUTI patients revealed that almost all CAZ %fT > MIC and AVI %fT > 0.5 mg/L values were close to 100% and unfavorable microbiologic outcomes (i.e., treatment failure) were relatively infrequent; thus, formal exposure-response modeling was not feasible.
Delafloxacin [24]	2017	ABSSSI	fAUC24/MIC Stasis: 9.3 1-log kill: 14.3		Neutropenic thigh, stasis and 1-log kill	Due to the limited number of clinical isolates for E coli and *P. aeruginosa* in Phase 3 clinical studies, clinical evidence appears insufficient to determine the breakpoints for *E. coli* and *P. aeruginosa.*
Meropenem-Vaborbactam [17]	2017	cUTI	%fT > MIC Static: 30% 1-log kill: 35% 2-log kill: 45%		Neutropenic thigh 1-log kill and 2-log kill	The rate of overall success in each group was >90%. Therefore, the analysis of outcomes for enterobacteriaceae demonstrated no obvious cutoff in MIC that discriminated between successes and failures.
Omadacycline [25]	2018	CABP, ABSSSI	AUC24/MIC 1-log kill: 33.3 S. pneumo 64.1 *E. coli*	Neutropenic 1-log kill 13.6 S. pneumo	Neutropenic thigh 1-log kill used to support ABSSSI, Neutropenic pneumonia 1-log kill used to support CABP	No targets derived from clinical data; however, success rates at higher MICs supported breakpoint decision in conjunction with non-clinical PKPD.
Plazomicin [26]	2018	cUTI, cIAI	AUC24/MIC Enterobacteriaceae Static: 24 1-log kill: 73 *K. Pneumo* Static: 30 1-log kill: 95	AUC24/MIC Enterobacteriaceae Static: 1.6 1-log kill: 6 *K. Pneumo* Static: 3.6 1-log kill: 9.5	Neutropenic thigh, stasis and 1-log kill	No exposure response was identified for cIAI or cUTI based on clinical data.
Cefiderocol [27]	2019	cUTI, HABP, VABP	%fT > MIC Static: 63.9% 1-log kill: 75.6%	%fT > MIC Static: 57.5% 1-log kill: 66.9%	Neutropenic thigh 1-log kill	Exposure response confirmed trend of efficacy in patients achieving 75% fT > MIC.
Imipenem-Relebactam [30]	2019	cUTI/cIAI	AUC24/MIC Relebactam Stasis: 4.8 1-log kill: 7.5		Neutropenic thigh, stasis and 1-log kill	Clinical PKPD targets were limited by insufficient data in the clinical trials.
Lefamulin [28]	2019	CABP		fAUC24/MIC Plasma 1-log kill: 2.97 2-log kill: 6.96 ELF 1-log kill: 30.4 2-log kill: 71.2	Plasma Neutropenic lung 1-log kill	Stratifying outcomes by MIC supported the breakpoint decision. Limited data at higher MICs.

**Table 2:** Comparison of epidemiologic cutoffs and breakpoints.

Antibiotic	Bacterial Species	Epidemiologic Cutoff (µg/mL)	Nonclinical PK/PD Cutoff (µg/mL)	Clinical Cutoff (µg/mL)	Overall Proposed Breakpoint (µg/mL)	Do FDA and Applicant Breakpoints Agree?
Ceftolozane-Tazobactam [29]	Enterobacteriaceae	2	4 ^1^	4	2	Not available
Ceftolozane-Tazobactam [29]	*P. aeruginosa*	4	4	1 ^2^	4	Not available
Dalbavancin ^3^ [21]	*S. aureus*	0.06	0.12–0.25	0.06	0.06 or 0.125	No (FDA lower)
Oritavancin ^3^ [22]	*S. aureus*	0.12–0.25	0.12	0.06	0.12	Yes
Ceftazidime-Avibactam [16]	*P. aeruginosa*	4–8	8	Not available	8	Not available
Tedizolid ^3^ [23]	*P. aeruginosa*	0.25–1	0.5	Not available	0.5	Yes
Delafloxacin [24]	*S. aureus*	0.25	0.25	Not available	0.25	Not available
Delafloxacin [24]	*E. coli*	4	0.25	Not available	0.25	Yes
Delafloxacin [24]	*P. aeruginosa*	>4	0.5	Not available	0.5	Yes
Meropenem-Vaborbactam [17]	*P. aeruginosa*	8	8	Not available	8	Yes
Meropenem-Vaborbactam [17]	Enterobacteriaceae	8	8	Not available	8	Yes
Omadacycline [25]	Enterobacteriaceae	>4	8	4	8	Not available
Omadacycline [25]	*S. aureus*	0.5	1	0.5	1	Not available
Plazomicin [26]	Enterobacteriaceae	2–4	1	Not available	1	Not available
Cefiderocol [27]	Enterobacteriaceae	4	4	Not available	2	Not available
Cefiderocol [27]	*P. aeruginosa*	2	4	Not available	1	Not available
Imipenem-Relebactam [30]	Enterobacteriaceae	2	4	2	1	Yes
Imipenem-Relebactam [30]	*P. aeruginosa*	0.5	4	4	2	Yes
Lefamulin [28]	*S. pneumoniae*	0.25	0.5	Not available	0.5	No
Lefamulin [28]	*S. aureus* (MSSA ^4^)	0.12	0.25	Not available	0.25	No

^1^ 1 µg/mL (tazobactam co-model), number in table represents cefazolin-only model. ^2^ Inadequate clinical data at higher MIC. ^3^ Epidemiologic cutoff not available and therefore approximated by MIC90 from microbiological review. ^4^ Methicillin-susceptible *Staphylococcus aureus.*

## Data Availability

All data presented in this manuscript are available to the public. The URLs are provided in the methods section, and specific documents are referenced below.
